# Glial Cell Contribution to Basal Vessel Diameter and Pressure-Initiated Vascular Responses in Rat Retina

**DOI:** 10.1167/iovs.16-20804

**Published:** 2017-01

**Authors:** Hui Li, Bang V. Bui, Grant Cull, Fang Wang, Lin Wang

**Affiliations:** 1 Department of Ophthalmology, The Tenth People's Hospital, Shanghai, Tongji University School of Medicine, Shanghai, China; 2 Devers Eye Institute, Legacy Research Institute, Portland, Oregon, USA; 3 Department of Optometry and Vision Sciences, University of Melbourne, Parkville, Victoria, Australia

**Keywords:** glial cell, calcium, intraocular pressure, retinal vasculature

## Abstract

**Purpose:**

The purpose of this study was to test the hypothesis that retinal glial cells modify basal vessel diameter and pressure-initiated vascular regulation in rat retina.

**Methods:**

In rats, L-2-aminoadipic acid (LAA, 10 nM) was intravitreally injected to inhibit glial cell activity. Twenty-four hours following injection, retinal glial intracellular calcium (Ca^2+^) was labeled with the fluorescent calcium indicator Fluo-4/AM (F4, 1 mM). At 110 minutes after injection, intraocular pressure (IOP) was elevated from 20 to 50 mm Hg. Prior to and during IOP elevation, Ca^2+^ and retinal vessel diameter were assessed using a spectral-domain optical coherence tomography/confocal scanning laser ophthalmoscope. Dynamic changes in Ca^2+^ and diameter from IOP elevation were quantified. The response in LAA-treated eyes was compared with vehicle treated control eyes.

**Results:**

L-2-Aminoadipic acid treatment significantly reduced F4-positive cells in the retina (LAA, 16 ± 20 vs. control, 55 ± 37 cells/mm^2^; *P* = 0.02). Twenty-four hours following LAA treatment, basal venous diameter was increased from 38.9 ± 3.9 to 51.8 ± 6.4 μm (*P* < 0.0001, *n* = 20), whereas arterial diameter was unchanged (from 30.3 ± 3.5 to 30.7 ± 2.8 μm; *P* = 0.64). In response to IOP elevation, LAA-treated eyes showed a smaller increase in glial cell Ca^2+^ around both arteries and veins in comparison with control (*P* < 0.001 for both). There was also significantly greater IOP-induced vasoconstriction in both vessel types (*P* = 0.05 and *P* = 0.02, respectively; *n* = 6 each).

**Conclusions:**

The results suggest that glial cells can modulate basal retinal venous diameter and contribute to pressure-initiated vascular responses.

Glial cells play critical roles in the maintenance of neuronal structure and function. Structurally, glia are rich with processes that surround blood vessels, weaving together to form an intercellular network of connections, thus placing them in an ideal position to communicate with cells involved in regulating vascular resistance.^1,2^ Glial cells have been identified as key intermediaries coordinating neurovascular coupling,^3–6^ a hyperemic response to increased neuronal activity (functional hyperemia). During neurovascular coupling, vasoactive signals arising from activated neurons or astrocytes influence local vessel tone and thus regulate regional blood flow to match the energetic needs of neuronal function.^6–9^ This regulatory process is thought to be a feed-forward signaling pathway, starting with increased neuronal activity and resulting in changes in arteriole diameter.^10^ It is known that triggering astrocyte calcium transients results in vasoconstriction of neighboring arterioles.^11^ Rosenegger et al.^12^ showed that glial cell–mediated effects on blood vessels were not affected by blocking neuronal activity using tetrodotoxin (voltage-gated sodium channel blocker). Indeed, chelation of astrocytic Ca^2+^ blunted changes in parenchymal artery vascular tone mediated by activation of mechanosensitive transient receptor potential cation channels (TRP) in brain slices.^13^ These outcomes suggest that in addition to their role in functional hyperemia, glial cells in the brain are involved in maintaining basal vascular tone. However, whether glia also contribute to basal vascular tone in retina vessels is unclear.

The capability for glia to modify vessel tone independent of neuronal activity also raises the possibility that glial cells may contribute to classical retinal blood flow autoregulation. Gordon et al.^14^ showed that, under high tissue oxygen conditions, triggering Ca^2+^ release in astrocytes resulted in vasoconstriction of adjacent vessels, whereas under low oxygen conditions, vasodilation was observed. The finding that vessel diameter can be modified in a bidirectional manner^15^ suggests that glia have the capacity to contribute to autoregulation in response to changes in the metabolic environment. There is some evidence that these same mechanisms also allow glia to participate in pressure-induced autoregulation.^13^ Hemodynamic challenge induced by elevating arterial perfusion pressure increased astrocyte Ca^2+^ levels in rat brain slices and in mice cortex in vivo. However, as the time course of in vivo Ca^2+^ changes could not be precisely determined, the relationship between pressure induced changes in Ca^2+^ and vessel diameter remains unclear.^13^

In our recent observation using in vivo imaging on rat retina, we showed that Ca^2+^ levels within perivascular glial cells around arteries and veins rapidly increase with intraocular pressure (IOP) elevation and closely follow the magnitude of IOP elevation. At the same time, peripapillary retinal artery diameter remained unchanged, whereas retinal veins significantly constricted. Although it was not possible to establish a temporal relationship between IOP-induced increases in glial Ca^2+^ and the vascular responses, venous constriction did not linearly follow the level of IOP elevation. This is contrary to an expected linear relationship between vein diameter and IOP magnitude if retinal veins did not have a basal tone and behaved as a simple Starling resistor.^16^ A recent study provides evidence that retinal veins without identifiable smooth muscle cells can be modulated by vasoconstricting and vasodilating agents.^17^ Thus, there is growing evidence that retinal veins are under some form of local autoregulation.

We reasoned that should glial cells be involved in the maintenance of basal vessel diameter (i.e., vascular tone) and pressure-initiated vascular regulation, one would expect that removing glial cell input should increase basal diameter and result in a greater reduction in vessel diameter in response to IOP elevation.

## Methods

### Animals

Thirty-six adult male Brown Norway rats (Charles River Laboratories, Inc., Wilmington, MA, USA) weighing 256 ± 23 (SD) g were used (control groups, *n* = 15; l-2-aminoadipic acid [LAA] groups, *n* = 21). Rats were maintained under a 12-hour light/12-hour dark cycle (1200 lux maximum, <100 lux inside the cage) with normal rat chow and water available ad libitum. All experimental methods and animal care procedures conformed to ARVO's Statement for the Use of Animals in Ophthalmic and Vision Research and were approved by the Legacy Institutional Animal Care and Use Committee.

### Anesthesia and Animal Preparation

Initial anesthesia was induced with an intramuscular (IM) injection of ketamine (75 mg/kg, Ketaset; Fort Dodge Animal Health, Fort Dodge, IA, USA) and xylazine (10 mg/kg, AnaSed; Lloyd, Inc., Shenandoah, IA, USA). A pad with circulating heated water (T/pump; Gaymar, Orchard Park, NY, USA) was used to maintain body temperature. A femoral vein and an artery were cannulated with polyethylene tubes for drug administration and blood pressure monitoring, respectively. Once the femoral vessels were cannulated, deep anesthesia was maintained for the remainder of the experiment via an intravenous infusion of sodium pentobarbital (∼10 mg/kg/h, Nembutal; Oak Pharmaceuticals, Lake Forest, IL, USA) using an infusion pump (Genie; Kent Scientific, Torrington, CT, USA). Prior to imaging, pupillary dilation was induced topically with tropicamide (0.5%; Alcon Laboratories, Inc., Fort Worth, TX, USA).

### Intravitreal Injection

Two regents were administered intravitreally: Fluo-4/AM (F-14217; Life Technologies, Carlsbad, CA, USA), a Ca^2+^ indicator dye (excitation/emission: 494/506 nm with Ca^2+^-bound form), and LAA (Sigma-Aldrich Corp., St. Louis, MO, USA), a gliotoxin. Fluo-4/AM (F4) solutions were freshly prepared by mixing 30 μL F4 (1 mM or 0.2 μg/μL) with 4.8 μL pluronic acid at 5.92 μg/μL (P-6867; Life Technologies). l-2-Aminoadipic acid is a glutamate analogue, which has been shown to be selectively taken up by glia leading to deactivation of glial cell function.^18^ Intravitreal application of the agent at a controlled dose causes loss of astrocytes and Müller cells, whereas the structure and function of retinal neurons remain normal.^19,20^ Stock LAA solution was prepared by dissolving 10 mg LAA into 1 ml 0.2 N hydrochloric acid (HCL). A 4-μL aliquot (diluted from the stock solution, 2.5 nM/μL) was drawn into a 10-μL Hamilton microsyringe (Hamilton Company, Reno, NV, USA). Following general anesthesia (intramuscular injection of ketamine [75 mg/kg], xylazine [10 mg/kg], and topical anesthesia with 0.5% proparacaine hydrochloride; Bausch & Lomb, Inc., Bridgewater, NJ, USA), the prepared solution was slowly injected into one randomly selected eye of each rat through the pars plana with the needle tip directed toward the posterior pole of the eye to avoid contact with the lens. Assuming a vitreous volume of 40 μL for adult rats, the final calculated concentration was 0.1 mM and 0.25 nM for F4 and LAA, respectively. Animals were monitored during recovery from anesthesia. Twenty-four hours after LAA injection, animals underwent surgery for femoral cannula implantation and subsequent imaging.

### Manometric Control of IOP

A 33-G needle connected to two reservoirs filled with balanced salt solution (Baxter, Deerfield, IL, USA) was inserted into the anterior chamber. The reservoirs were set at two precalibrated heights, which allowed rapid switching between 20 and 50 mm Hg, as measured using a pressure transducer (MX860; Medex, Inc., Carlsbad, CA, USA) monitored via a data acquisition system (LAB-TRAX4-24T; WPI, Sarasota, FL, USA).

### Retinal Glial Cell Ca^2+^ and Vessel Diameter Imaging

Following surgical cannula placement under anesthesia, rats were placed on a stage in front of a spectral-domain optical coherence tomography/confocal scanning laser ophthalmoscope (SDOCT/cSLO, Spectralis; Heidelberg Engineering, Heidelberg, Germany). Custom-made rigid gas-permeable contact lenses (3.5-mm posterior radius of curvature, 5.0-mm optical zone diameter, and +5.0-diopter back vertex power) were placed on both eyes. The time course of changes in F4 fluorescence intensity (representing relative Ca^2+^ in glial cells) and retinal vessel diameter during acute IOP elevation were recorded by fluorescence angiograph mode (cSLO-FM) and infrared reflection mode (cSLO-IR) of the cSLO/SDOCT, respectively. All measurements were undertaken across a 30° field centered at the optic disc at a frame rate of 10 Hz.

The effect of LAA on basal vessel diameter was assessed by imaging immediately before and 24 hours after intravitreal LAA injection. For this purpose, we used the 15° circular OCT B-scan centered around the optic disc. The use of the OCT B-scan allows the “follow-up” mode to be used, which is a feature not available with the cSLO in infrared (cSLO-IR) mode. This follow-up feature allows the same retinal region to be scanned before and after LAA treatment, thus minimizing error in vessel diameter estimates arising from intersession differences in imaging location.

### Analysis of Calcium Fluorescence Intensity and Vessel Diameter

#### Ca^2+^ and Vessel Diameter Assessment.

Changes in Ca^2+^ and vessel diameter induced by IOP elevation were quantified using Fiji (ImageJ v2.0.0; National Institutes of Health, Bethesda, MD, USA),^21^ an open source platform for biological image analysis offline. In brief, image sequences acquired immediately prior to and during acute IOP elevation were registered (StackReg, ImageJ; National Institutes of Health) and averaged to produce a single image every 2 seconds. This set of averaged images was combined into a single stack and registered to allow the same regions of interest to be analyzed across time. Regions of interest were selected along arteries and veins starting at the optic disc margin and ending at one disc diameter distance from the margin. Vessel diameter was analyzed at 0.5 disc diameters from the disc margin, in the middle of the region of interest used for Ca^2+^ quantification. In each retina, Ca^2+^ and vessel diameter were measured from three to five arteries or veins. For each vessel, changes induced by IOP elevation were expressed as a percentage of the baseline measured immediately prior to IOP elevation. Group data for each vessel type were then averaged.

#### Vessel Diameter Quantification From OCT Images.

Masked observers measured vessel width from OCT circle scans using the ImageJ *Diameter* plugin (National Institutes of Health).^22^ This plug-in measures the width at half-maximum contrast. A line selection was first drawn on the selected vessel shadow perpendicular to retinal surface at the level of the outer nuclear layer. The measurement line was drawn to be at least twice the width of the blood vessel shadow at that location. This was repeated five times, and the output was averaged to return a single vessel diameter at each time point.

### Experiment Design

#### Basal Vessel Diameter.

Twenty rat eyes received an intravitreal injection of LAA. To test whether inactivated glial cells modify basal vessel diameter, imaging was undertaken immediately before and 24 hours following LAA injection.

#### Pressure-Initiated Responses.

We assessed the effect of IOP elevation in control rat eyes (Ca^2+^ group, *n* = 6; Ø group, *n* = 7) and those treated 24 hours earlier with LAA (LAA Ca^2+^ group, *n* = 6; LAA Ø group, *n* = 7) for quantification of glial Ca^2+^ or vessel diameter. For those animals in the Ca^2+^ group, 5 μL F4 solution was injected intravitreally into the eye under isoflurane anesthesia. Approximately 110 minutes after F4 injection, rats were anesthetized with ketamine:xylazine for femoral cannula placement surgery and in vivo imaging (pentobarbital). The F4-injected eye had an anterior chamber cannula inserted to allow for IOP elevation from 20 to 50 mm Hg. Imaging in cSLO-FM mode to track Ca^2+^ began 10 seconds before IOP onset and continued for 45 seconds during IOP elevation for a total time of approximately 1 minute. For the Ø groups, the same imaging protocol was used with the device placed in cSLO-IR mode. The following comparisons were made: (1) vessel diameter in eyes before and after LAA treatment (at baseline IOP of 20 mm Hg), (2) dynamics of Ca^2+^ and vessel diameter changes from baseline at every 2 seconds during IOP elevation, and (3) dynamics of Ca^2+^ or vessel diameter changes in LAA-treated and control eyes (control Ca^2+^ group and control Ø group).

Before and after manometric IOP elevation, IOP in the tested eye was measured using a rodent tonometer (TONOLAB, Vantaa, Finland) to confirm the set IOP levels. As blood pressure is known to affect blood vessel diameter, blood pressure was monitored continuously via the femoral artery. Blood pressure throughout the imaging run was averaged for each animal. Group average blood pressure (±SD) was found to be similar between the experimental groups and control (LAA group: 81.4 ± 2.7 mm Hg; control group: 84.2 ± 1.5; *P* = 0.36, unpaired *t*-test).

It is important to rule out the possibility that the LAA dose used did not directly affect the contractility of vessels. This would lead to a change in vessel diameter not related to inhibition of glial input, leading to potentially erroneous interpretation of glial cell contributions to retinal vessel tone. Thus, we examined the contractility of vascular smooth muscle by using endothelin-1 (ET-1), a potent vasoconstrictor. We assessed the effect of intravitreal injection of ET-1 in control rat eyes (*n* = 6) and those treated 24 hours earlier with LAA (*n* = 6). Vessel diameter was imaged using the OCT circle scan, as described above, prior to and 60 minutes following intravitreal injection of ET-1 (5 pM; Sigma-Aldrich Corp.). The effect of ET-1 (%, relative to baseline) on vessel diameter in control and LAA-treated eyes was quantified using ImageJ (National Institutes of Health) as described above.

### Immunohistochemistry

The effect of LAA on F4 uptake by glial cells was evaluated 24 hours after intravitreal injection of LAA. Retinae were isolated ∼110 minutes after F4 injection and immediately imaged without fixation (to avoid F4 bleaching) using a fluorescence microscope (Leica, Wetzlar, Germany). Across a retinal area of approximately 6 mm^2^, the number of cells loaded with F4 were counted by a masked observer. The average density of F4 positive cell (#/mm^2^) was compared between eyes with and without LAA treatment.

LAA-treated (*n* = 6) and control eyes (*n* = 4) were fixed in 4% paraformaldehyde and cut into 12-μm cryo-sections for immunohistochemistry to evaluate the status of retinal glial cells. Sections were first incubated in Triton X-100 0.03% for 15 minutes and then in blocking serum (2% goat serum and 2% bovine serum albumin) for 1 hour followed by overnight (4°C) application of a rabbit primary antibody to glial fibrillary acidic protein (GFAP, 1:400; Sigma-Aldrich Corp.) and mouse primary antibody to glutamate synthase (GS, 1:400; Abcam, Cambridge, MA, USA). After three washes in 0.01 M PBS, each for 10 minutes, retinal sections were incubated overnight (4°C) with goat anti-mouse and goat anti-rabbit secondary antibodies conjugated with AlexaFluor 488 (1:200) and 555 (1:400; Life Technologies, Grand Island, NY, USA), respectively. Tissues were washed with PBS and imaged using a confocal fluorescence microscope (DMi8, Confocal system TCSSPE; Leica).

### Statistics

Data are presented as mean ± SD unless otherwise indicated. Statistical analysis was performed using Prism 6 (GraphPad Software, Inc., La Jolla, CA, USA). When comparing data sets collected from the same group of animals over time, 1-way repeat-measures ANOVA (RM-ANOVA) was used, with a post hoc comparison performed using the Dunnett's test. Comparisons between groups with and without LAA treatment used 2-way RM-ANOVA. For comparison between group means, either a paired or unpaired Student's *t*-test was used once normality was established using the D'Asgostino Pearson normality test.

## Results

### Effect of LAA on Retinal Glial Cells

**Figure 1:**
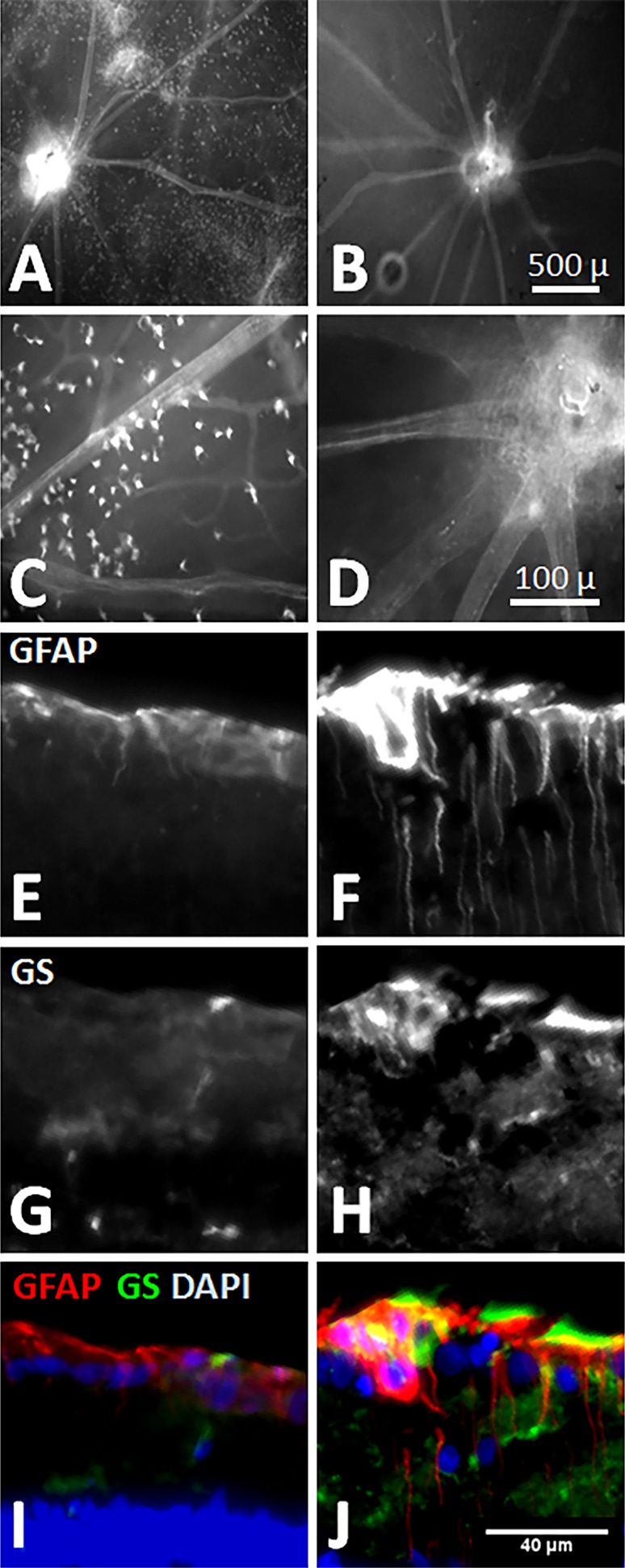
Effect of the gliotoxin LAA on retinal glial cells (*right*) compared with control (*left*). (**A**, **C**) Fluorescence microscopy of F4-loaded cells in freshly isolated retina from a representative control eye. Note the punctate staining indicative to F4 uptake. (**B**, **D**) F4-positive cells are sporadic in LAA treated eyes compared with control eye (**A**, **C**). (**E**, **F**) Retinal cryosections stained for GFAP. Note the radially distributed glial cell processes across the retina, in addition to enhanced immunoreactivity in the surface of retina (*top*: vitreous side) in the LAA-treated eye. (**G**, **H**) In the same eyes shown in **E** and **F**, GS immunoreactivity is increased following LAA treatment. (**I**, **J**) Overlay of GFAP (*green*), GS (*green*), and DAPI (*blue*) shows that both Müller cell endfeet and astrocytes are activated in the retinal nerve fiber layer in the LAA-treated eye. Same scale (*bottom right*) applies to all microphotographs from **E** through **J**.

Figure 1A shows fluorescence images of freshly isolated retina from a control eye after 30 minutes of F4 incubation. The punctate staining apparent in Figure 1A was absent from the LAA-treated retina shown in Figure 1B. At higher magnification, the astrocytes cell bodies that have taken up F4 could be seen in control (Fig. 1C) but not LAA-treated retinas (Fig. 1D). Cell counts show that in retinas 24 hours after intravitreal injection of LAA, the number of F4-positive cells was significantly reduced compared with control eyes (16 ± 20 [mean ± SD, *n* = 6] vs. 55 ± 37 cells/mm^2^ [*n* = 4] respectively; *P* = 0.02, unpaired *t*-test). It should be noted that punctate F4 fluorescent cells seen in isolated retina might not be obvious in in vivo images due to (1) poorer image resolution in vivo and (2) difference between in vivo and isolated retinas in terms of Ca^2+^ concentration and distribution within glial cells body and processes.

**Figure 2:**
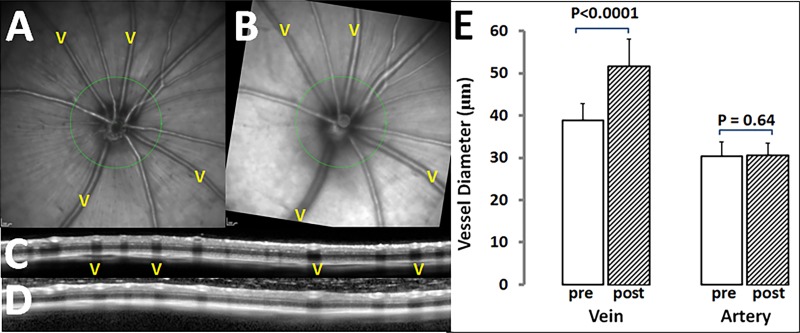
Effect of LAA on retinal vessel diameter measured using OCT. Confocal scanning laser ophthalmoscope–IR images of a rat eye before (**A**) and 24 hours after intravitreal injection of LAA (**B**); the latter shows that veins (v) are wider. (**C**, **D**) Optical coherence tomography peripapillary circular scan images cross arteries and veins (*green circles* in **A** and **B**, respectively) before and after LAA treatment. The first “v” in **C** and **D** corresponds to the vein at 10 o'clock in images **A** and **B**. (**E**) Average retinal vein and artery (*n* = 20 eyes each) diameters before (*open columns*) and 24 hours after LAA treatment (*hatched columns*). For each eye, vessel diameter is the average of three to five arteries or veins.

**Figure 3:**
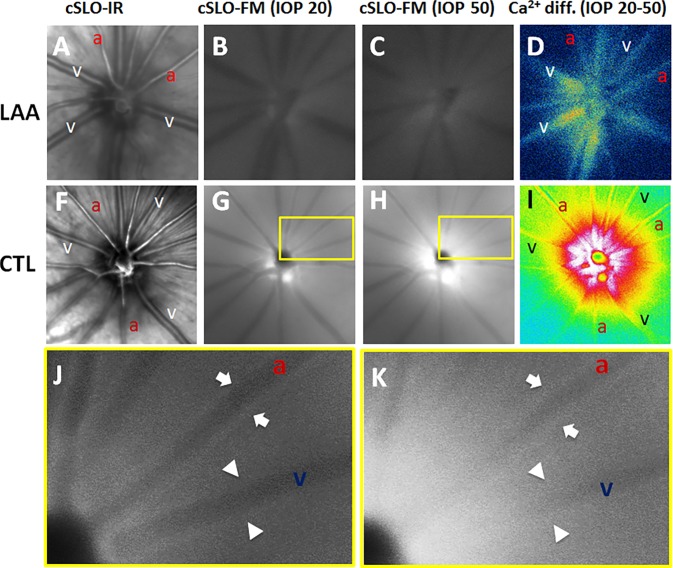
Intraocular pressure–induced changes in Ca^2+^ in a representative LAA-treated (*top*) eye and a control (*middle*) eye. (**A**, **F**) Confocal scanning laser ophthalmoscope–IR images with labels indicating arteries (a) and veins (v). (**B**, **G**) Confocal scanning laser ophthalmoscope–FM images before acute IOP elevation (baseline IOP, 20 mm Hg). (**C**, **H**) Confocal scanning laser ophthalmoscope–FM images 45 seconds after IOP elevation (IOP, 50 mm Hg). (**D**, **I**) Ca^2+^ intensity difference between baseline and final images derived by subtraction (warmer colors indicate a greater change in Ca^2+^ intensity). (**J**, **K**) Close-up of *boxed areas* in **G** and **H**, respectively. *Arrows* indicate the Ca^2+^ along edges of the artery and *arrowheads* indicate the Ca^2+^ along edges of the vein. Notice that the increased Ca^2+^ intensity along the vessels in **K** reduces the vessels shadows in **J**.

Additionally, there is variability in F4 cell density, which may have arisen from a number of sources. Although care was taken to standardize intravitreal injection, there may be variability in how much F4 reached the retina. Additionally, the time taken between tissue harvest and imaging and the potential for bleaching might also have introduced variability.

In comparison to control eyes, GFAP immunoreactivity in all six LAA-treated eyes was stronger within the retinal nerve fiber layer, where both astrocytes processes and the Müller cells endplates locate. The GS staining was colocalized with GFAP immunoreactivity within both the retinal nerve fiber layer and the inner nuclear and inner plexiform layers that are rich in Müller cell processes (Fig. 1J). These results suggest that LAA affected both astrocytes and Müller cells in the retina. Parapapillary retinal nerve fiber layer thickness in LAA-treated eyes was significantly increased as measured by OCT (pre- versus post-: 45.2 ± 3.3 vs. 54.6 ± 4.7 μm; *P* < 0.0001). DAPI (4′,6-diamidino-2-phenylindole) staining showed no remarkable change in cells population.

## Effect of LAA on Basal Vessel Diameter

Twenty-four hours after LAA injection, veins were wider, whereas arteries showed little change. Figure 2 shows the effect of LAA treatment on basal vessel diameter in a representative eye (Figs. 2A, 2B). Figures 2C and 2D compare circular OCT scans taken at 0.5 disc diameters form the edge of the optic nerve. Inspection of the vessel shadows shows that the diameters of veins were larger following LAA treatment. The group average vein diameter was increased from 38.9 ± 3.9 μm (baseline) to 51.8 ± 6.4 μm 24 hours after LAA injection (*P* < 0.0001, paired *t*-test; *n* = 20). Arterial diameter showed no significant change at 24 hours following LAA injection (from 30.3 ± 3.5 to 30.7 ± 2.8 μm; *P* = 0.64; *n* = 20).

### Effect of LAA on IOP-Induced Changes in Paravascular Ca^2+^

Figure 3 illustrates the effect of IOP elevation on F4 fluorescence intensity in a representative LAA-treated and a control eye. Change in F4 intensity was much smaller for the LAA-treated eye (difference between Figs. 3B and 3C) compared with the control (difference between Figs. 3G and 3H). This is better appreciated via the difference images shown in Figures 3D and 3I for the LAA-treated and control eyes, respectively.

**Figure 4:**
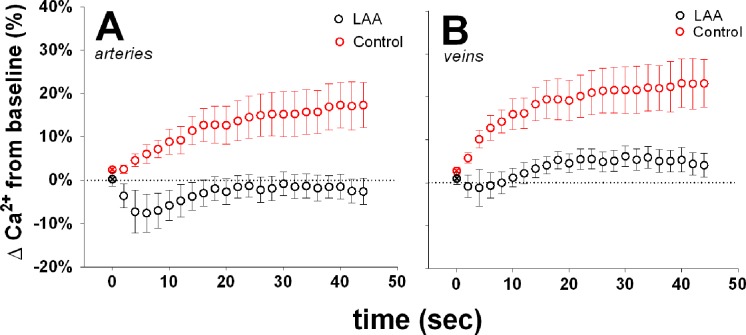
Effect of acute IOP elevation on Ca^2+^ levels surrounding retinal arteries and veins in control (*n* = 6) and LAA-treated eyes (*n* = 6). Change in Ca^2+^ intensity was expressed relative to baseline (⊗, first symbol of each data series) and plotted as a function of time following IOP elevation from 20 to 50 mm Hg. (**A**) Intraocular pressure elevation induced a significant increase in Ca^2+^ around arteries in control (*red circles*). There was significantly less change in LAA-treated eyes (*black circles*). (**B**) Glial Ca^2+^ around retinal veins was significantly increased with IOP elevation in control eyes. This response was significantly smaller in LAA-treated eyes. *Error bars* denote ±SEM.

Figure 4 shows the changes in Ca^2+^ intensity as a function of time during IOP elevation from 20 to 50 mm Hg. In control eyes, acute IOP elevation induced a significant increase in Ca^2+^ intensity around both arteries (Fig. 4A; 1-way ANOVA, *P* < 0.001) and veins (Fig. 4B; *P* < 0.001). At 44 seconds following IOP elevation, Ca^2+^ levels were increased by 14 ± 11% in arteries and 20 ±12% in veins (mean ± SEM). In comparison, LAA-treated eyes showed a significantly smaller IOP-induced Ca^2+^ response around both arteries and veins (2-way RM-ANOVA interaction effect, *P* < 0.001 for both).

### Effect of LAA on IOP-Induced Changes in Vessel Diameter

**Figure 5:**
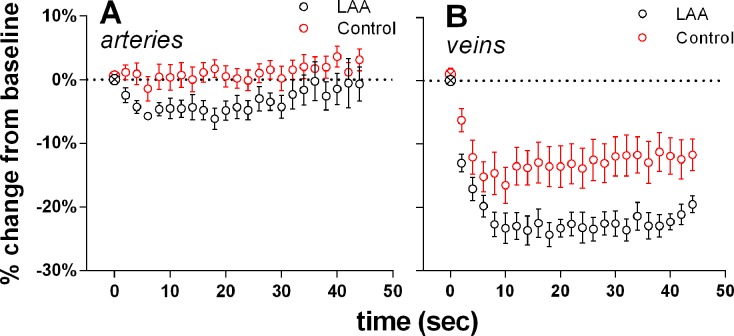
Effect of IOP elevation on arterial and venous diameter in control (*n* = 7) and LAA-treated eyes (*n* = 7). Change in diameter was expressed relative to baseline (⊗, first symbol of each data series) and plotted as a function of time after IOP elevation from 20 to 50 mm Hg. (**A**) Intraocular pressure elevation induced a significant decrease in arterial diameter in LAA-treated eyes (*P* = 0.05; *black circles*) but not in control eyes (*P* = 0.34; *red circles*). (**B**) Venous diameter was significantly reduced in both groups (*P* = 0.01 for both). However, IOP-induced attenuation of venous diameter was significantly greater in LAA eyes compared with controls (*P* = 0.02). *Error bars* denote ±SEM.

For retinal arteries, acute IOP elevation caused significant vasoconstriction from baseline in the LAA-treated group (*P* = 0.05) but not in the control group (*P* = 0.19). For retinal veins, IOP elevation caused significant vasoconstriction in the LAA-treated group (*P* < 0.0001). The constriction in LAA-treated eyes was significantly greater than the control group (*P* = 0.02, 2-way RM-ANOVA; Fig. 5).

### Effect of LAA on Vessel Contractility Induced by ET-1

**Figure 6:**
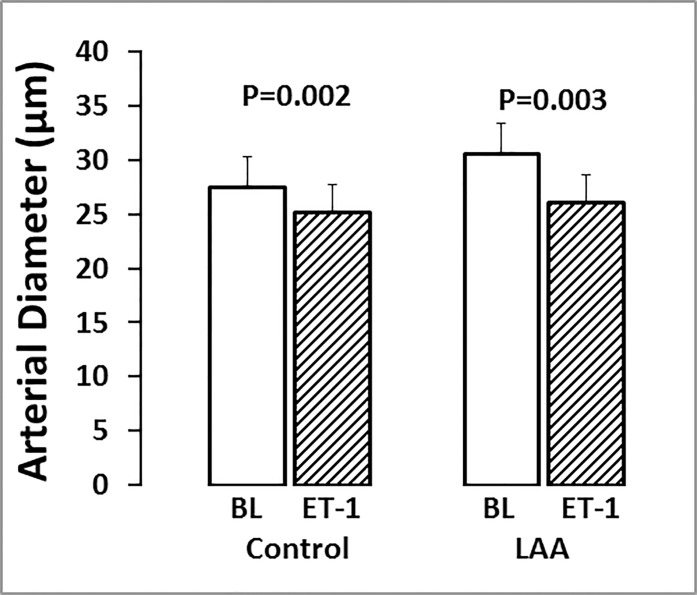
Effect of endothelin-1 on vessel diameter in control and LAA-treated eyes. Retinal arterial diameter (mean ± SD) at baseline (BL, *open columns*) and 60 minutes after intravitreal ET-1 injection (*hatched columns*) in six control and six LAA-treated eyes. In each eye, vessel diameter was averaged from three to five arteries.

To consider whether LAA had directly impaired or damaged the function of smooth muscle cells, the contractility of arteries in response to intravitreal ET-1 injection was assessed in eyes with and without LAA treatment (*n* = 6, each). Approximately 60 minutes after ET-1 injection, arterial diameter decreased by −7.9 ± 3.2% in control eyes (*P* = 0.002) and −11.6 ± 9.0% in LAA eyes (*P* = 0.003; Fig. 6).

## Discussion

After inhibiting retinal glial activity in rat eyes, we found that the diameter of veins measured near the optic nerve was significantly increased, which was not the case for arteries at the same location. In LAA-treated eyes, acute IOP elevation produced significantly less increase in glial Ca^2+^, but significant more constriction of both arteries and veins compared with control eyes. These results support the idea that glial cells contribute to basal retinal vascular tone in particularly veins. In addition, glial cells also appear to modify the dynamic response to arteries and veins to changes in perfusion pressure.

To modulate vascular resistance and the blood flow, blood vessels exhibit some degree of constriction or basal tone.^23^ In arteries, vascular tone is largely determined by a balance of vasoconstricting and vasodilating factors acting on smooth muscle cells. Interestingly, Kim et al.^13^ showed that chelating Ca^2+^ and reducing the activity of astrocytes in rat brain slices increased parenchymal artery diameter. Following inhibition of retinal glial cells using LAA, we also found an increase in vessel diameter. In particular, glia appear to make a contribution to the basal vascular diameter of retinal veins but not arteries. Kim et al.^13^ used an in vitro brain slice approach; this isolated system, along with the more complete inhibition of astrocytes by the chelating agent compared with LAA treatment, may account for our inability to find a glial cell contribution to arterial basal tone. Differences in oxygen and acidity between in vivo and in vitro preparations may also influence relative arterial tone^24^ and contribute to differences between the finding of Kim et al.^13^ and our finding. Finally, there may be differences in glial contribution to basal vessel tone between retina and brain.

Our finding of substantial (+39%) increases in venous diameter following LAA treatment is at first perplexing as retinal veins are known to be free of SMCs. However, it has been shown that retinal veins have a rich coat of glial cells.^25^ Yu et al.^17^ recently showed that ET-1 and adenosine could effectively induce vasoconstriction and vasodilation in porcine retinal veins without any identifiable smooth muscle cells (SMCs). Thus, the removal of glial activity on the retinal vein via LAA may account for the increased venous diameter and suggest the possibility that glial cells are able to produce a vasomotor effect independent of SMCs.

In addition to modifying basal retinal vessel diameter, LAA treatment also significantly attenuated IOP-induced changes in glial Ca^2+^ and exaggerated changes in vessel diameter. As shown in Figure 4, acutely increasing IOP from 20 to 50 mm Hg (a 30-mm Hg reduction in perfusion pressure) elicited rapid increases in perivascular glial cell Ca^2+^ along both retinal arteries and veins. After LAA treatment, the IOP-induced Ca^2+^ response was significantly blunted. Without LAA treatment, arteries maintained their diameter during IOP elevation, whereas venous diameter reduced during the IOP elevation. Veins in LAA-treated eyes showed a much greater reduction in diameter in response to IOP elevation compared with control. This pattern of change in diameter suggest that glial cell inhibition reduced the capacity of the large retinal vessels to resist IOP induced compression and act more like Starling resistors. Thus, these data provide evidence that retinal glial cells are capable of sensing the reduction in perfusion pressure and contribute to the maintenance of vessel diameter.

We explored the potential for toxic effects of LAA that might weaken SMCs contractility, thereby increasing vessel diameter independent of any glial cell effect. The robust vasoconstriction following injection of ET-1 in both control and LAA-treated eyes (Fig. 6) shows that arterial SMCs retained the capacity to constrict. The same finding was reported recently by using ex vivo porcine retina.^26^ Previous studies used LAA at some twofold higher concentration than that used in the current study without any detectable damage to retinal structure or function as examined using histology and electroretinography, respectively.^20^ Higher concentrations of LAA have also been shown to be nontoxic to in vitro endothelial cells and pericytes.^27^ It should be noted that, because glial cells provide structural and metabolic support for neurons, depletion of retinal glial cells may cause neuronal malfunction, which in turn affects their interaction with the vasculature. However, Song et al.^20^ showed that 24 hours after intravitreal injection of LAA, retinal function remained normal as examined using electroretinography. Thus, our finding of a significant increase in basal vessel diameter, along with the greater reduction in vessel diameter during IOP elevation in LAA-treated eyes, is likely to have arisen from loss of glial cell activity. A limitation of using LAA to disable retinal glial cells is the potential for an incomplete removal of glial cell contributions. In most retinas treated with LAA, we observed F4 intensity was reduced using cLSO-FM. However, the background fluorescence was not completely removed, and in some cases, it remained relatively strong (e.g., Fig. 3B). Imaging of freshly isolated LAA-treated retinas confirmed that there were fewer F4-positive cells, but not a complete loss. Thus, it is likely that functional glial cells remained due to an incomplete effect of LAA. It is also not possible to rule out the possibility that F4 might have been taken up by nonglial cells. Nevertheless, our observation of a severely blunted Ca^2+^ response to IOP elevation is consistent with the majority of glial cells being inactivated by LAA as evidenced by the reduction in F4 fluorescent cell density (Fig. 1).

In summary, our study shows that retinal glial cells contribute to the basal vascular diameter and dynamic vascular response to perfusion pressure lowering of the larger vessels in the rat retina. Retinal veins appear to be influenced by glial cells to a greater extent than arteries. Thus, glial cells may play a more important role in pressure-initiated blood flow autoregulation than previously thought.
